# Circadian rhythm phase shifts and endogenous free-running circadian period differ between African-Americans and European-Americans

**DOI:** 10.1038/srep08381

**Published:** 2015-02-11

**Authors:** Charmane I. Eastman, Christina Suh, Victoria A. Tomaka, Stephanie J. Crowley

**Affiliations:** 1 Biological Rhythms Research Laboratory, Department of Behavioural Sciences, Rush University Medical Center, Chicago, Illinois 60612, USA

## Abstract

Successful adaptation to modern civilization requires the internal circadian clock to make large phase shifts in response to circumstances (e.g., jet travel and shift work) that were not encountered during most of our evolution. We found that the magnitude and direction of the circadian clock's phase shift after the light/dark and sleep/wake/meal schedule was phase-advanced (made earlier) by 9 hours differed in European-Americans compared to African-Americans. European-Americans had larger phase shifts, but were more likely to phase-delay after the 9-hour advance (to phase shift in the wrong direction). The magnitude and direction of the phase shift was related to the free-running circadian period, and European-Americans had a longer circadian period than African-Americans. Circadian period was related to the percent Sub-Saharan African and European ancestry from DNA samples. We speculate that a short circadian period was advantageous during our evolution in Africa and lengthened with northern migrations out of Africa. The differences in circadian rhythms remaining today are relevant for understanding and treating the modern circadian-rhythm-based disorders which are due to a misalignment between the internal circadian rhythms and the times for sleep, work, school and meals.

Phase shifts in endogenous human circadian rhythms are required for adaptation to jet travel across multiple time zones, for physiological adaptation to night shift work with daytime sleep and for adaptation to early morning shift work which necessitates early awakenings. Extreme night owls, people with the delayed sleep phase disorder, and older adolescents who have difficulty waking for school could benefit from phase shifting the circadian system earlier to adapt to society's demands. In all of these conditions, the times for sleep, work, school and meals are misaligned with the internal circadian rhythms. The circadian misalignment resulting from shifting sleep times earlier on workdays or school days compared to weekends or days off, known as social jet lag, is associated with smoking, obesity and depression^1^,^2^,^3^. Shift work, which produces massive circadian misalignment, is classified as a probable carcinogen and is a risk factor for obesity and cardiovascular, gastrointestinal and metabolic diseases (see Smith and Eastman^4^ for a review).

Laboratory studies over about the last 40 years have shown that human circadian rhythms phase shift very slowly in response to a large, abrupt phase shift (e.g. Refs. 5,6,7,8,9,10,11,12,13) or gradual phase shifts (e.g. Refs. 14,15,16) of the sleep schedule, although appropriately timed bright light or melatonin can accelerate the phase shift. In two previous studies^17^,^18^, we used bright light exposure and a gradually shifting sleep (dark) schedule to phase-shift the human circadian clock. When the data from these two studies were analyzed according to race/ethnicity^19^, there were unexpected differences between African-Americans and European-Americans. In the study designed to produce advances (shifts earlier in time)^17^, the African-Americans phase-advanced more than the European-Americans, but in the study designed to produce delays (shifts later in time)^18^, there was a suggestion that the African-Americans phase-delayed less than the European-Americans. One factor that could account for these differences is the free-running circadian period (also called the endogenous or intrinsic period (τ)) since, theoretically, a shorter underlying period should facilitate advances and a longer period should facilitate delays^20^. We had been measuring the free-running circadian period for several years as part of other studies^21^,^22^,^23^,^24^. In retrospective analyses of these data^19^,^25^, we found that African-Americans had a shorter circadian period than European-Americans. This might explain any differences in circadian rhythm phase shifts.

These findings were evidence for racial differences in the human circadian system. We did not, however, have as many African-Americans as European-Americans in our samples of circadian period. In our largest data set^25^, there were 20 African-Americans and 55 European-Americans, but we only had 3 African-American men. In our phase shifting studies, the sample sizes were also very small, with only 2 African-Americans in one group. Furthermore, the phase shifting data and the circadian period data were not collected from the same subjects so we could not show a direct link between phase shifts and circadian period. Finally, only participant's self-reported race/ethnicity was available, and we did not know about the race-ethnicity or their parents and did not have genetic analyses of ancestry. Therefore, the aims of the current prospective study (see the protocol in Fig. 1) were to examine phase shifts in response to a large (9-h), abrupt phase advance of 24-hour zeitgebers (24-hour time cues), and to measure the free-running circadian period in African-Americans compared to European-Americans with equal numbers in each group and equal numbers of men and women. Furthermore, we collected participant-reported race of parents and grandparents and buccal DNA samples to estimate genetic ancestry.

To our knowledge, this is the first study to compare the phase shifts of humans from different races, in this case African-Americans and European-Americans, after a large abrupt phase shift of sleep as would be experienced after crossing many time zones or working night shifts. To our knowledge, this is also the first study to show the relationship between phase shifts and circadian period within a species, although the relationship has been shown across species^20^. We do not know of any other studies of racial/ethnic differences in circadian rhythms, or any studies relating human circadian rhythms to genetic ancestry.

## Results

Table 1 shows that the % European ancestry and the % Sub-Saharan African ancestry was very different in the African- Americans compared to the European-Americans, as expected. We did not obtain genetic ancestry results for one African-American because there was not enough material in the buccal swab, so these results are for 18 African-Americans. For the African- Americans, the % Sub-Saharan African ancestry ranged from 58 to 98, and the % European ancestry ranged from 0 to 32%. For the European-Americans, the % European ancestry ranged from 80 to 100, and the % Sub-Saharan African ancestry ranged from 0 to 12. Each subject's % ancestry in each category had a 95% confidence interval with a lower and an upper limit. For the African-Americans the mean ± SD lower and upper limits were 64.1 ± 12.5 and 85.6 ± 8.9 for % Sub-Saharan African and 5.2 ± 6.3 and 27.2 ± 12.4 for % European. For the European-Americans, it was 75.4 ± 11.3 and 96.4 ± 6.0 for the % European and 0.2 ± 1.0 and 12.7 ± 6.3 for the % Sub-Saharan African.

### Free-Running Circadian Period (τ)

The average free-running period (τ) of the 36 subjects was 24.21 ± .23 h and ranged from 23.79 to 24.77 h. There was overlap in the distributions of circadian periods for African and European-Americans (Fig. 2), but the difference between the groups was statistically significant [t = 4.784, p < 0.001] (Table 2). Only African-Americans had circadian periods <24.00 h. Four of the 19 African-Americans (21%), but none of the European-Americans had circadian periods <24.00 h. The proportion with circadian period <24.00 h differed between groups (Chi-Square = 4.026, p = 0.045).

Longer circadian periods were associated with a greater percent of European ancestry and a smaller percent of African ancestry. When the European-Americans and African-Americans were combined (N = 35, 18 African-Americans + 17 European-Americans), a positive correlation was seen between circadian period and European ancestry (r = +.63, p < 0.001, 2-tailed) and a negative correlation was seen between circadian period and Sub-Saharan African ancestry (r = −.64, p < 0.001). When the groups were considered separately, producing less variation in both variables, only the association within the African-American group remained significant. For African-Americans, individuals with a greater proportion of European ancestry had longer circadian periods (r = +.49, p = 0.037, N = 18).

There were no sex differences in circadian period; however, a trend for a sex-by-race interaction [F(1,32) = 4.106, p = 0.051] was seen suggesting that the differences between men and women were the opposite in African-Americans compared to European-Americans. For European-Americans, women had a descriptively shorter circadian period than men (24.28 ± .16 vs 24.43 ± .24 h), whereas for African-Americans, men had a descriptively shorter circadian period than women (24.03 ± .16 vs 24.12 ± .12 h).

### Phase Angle of Entrainment during Baseline

The phase angle of entrainment – the temporal relation between the internal circadian clock and external zeitgebers (time cues) – was associated with the endogenous free-running circadian period (Fig. 3). Subjects with shorter circadian periods had earlier circadian rhythms (earlier dim light melatonin onsets (DLMOs)) relative to dark (relative to bedtime = lights out), and subjects with longer circadian periods had later circadian rhythms relative to dark (r = +.58, p = 0.009 for the African-Americans (N = 19); r = +.50, p = 0.039 for the European-Americans (N = 17); r = +.52, p = .001 for both (N = 36)).

### Phase Shifts to 9-hour Phase Advance of Zeitgebers

The phase shifts of the circadian clocks (marked by the DLMOs) after the 9-h advance are shown in Figure 4. Most of the African-Americans phase advanced, but about half the European-Americans phase delayed; they shifted in the wrong direction, which is known as antidromic re-entrainment. The number who advanced or delayed more than 0.5 h (more than our saliva sampling frequency for the DLMO which was every 0.5 h) is shown in Table 2. This difference in phase shift direction (advance vs delay) between African and European-Americans was statistically significant (Chi-Square = 4.386, p = 0.036). African and European-Americans did not differ in phase shift. Some European-Americans, however, had the largest phase shifts regardless of direction. When the absolute magnitude of the phase shifts was compared (instead of positive numbers for advances and negative numbers for delays), European-Americans phase shifted more than African-Americans (Table 2).

The phase shift of the circadian clock due to the 9-h advance was associated with the free-running circadian period (Fig. 5). Subjects with shorter circadian periods had larger phase advances of the DLMO, and subjects with longer circadian periods had larger phase delays (r = −.58, p = 0.015 for the European-Americans; r = −.40, p = 0.087 for the African-Americans; and r = −.56, p < 0.001 for both).

We also examined phase shifts while accounting for each individual's circadian period by subtracting the change in phase that would be produced by a free-run from days 10 to 14 from the standard phase shift values presented above (which were the changes in DLMOs from days 10 to 14). These “corrected” phase shift values produced results that were similar to those from the standard phase shift values. When plotted as in Figure 4, more European-Americans than African-Americans shifted in the wrong direction, and some European-Americans had the largest phase advances and the largest phase delays. When the absolute values of the corrected phase shifts were compared between African-Americans and European-Americans the results were similar to those shown in Table 2 (which shows standard phase shift values). European-Americans had larger corrected phase shifts than African-Americans (2.5 ± 1.6 h vs 1.7 ± 0.8 h, p < 0.0001 by t-test).

A correlation matrix including baseline bedtime, baseline phase, baseline phase angle of entrainment, phase shift, and free-running period is included in Supplementary Table S1.

## Discussion

This was the first study designed to examine differences in circadian rhythms among human races. We found that African-Americans had shorter endogenous free-running circadian periods than European-Americans, and that circadian period (τ) was correlated with the percent African and European genetic ancestry. After a large, 9-hour, abrupt phase advance shift of zeitgebers (time cues), European-Americans were more likely to phase delay, to phase shift in the wrong direction, than African-Americans. We found a correlation between the phase relationship that the circadian clock assumes within the 24-hour day (called the phase angle of entrainment) and the free-running circadian period. We also found a correlation between phase shifts after the large, abrupt phase shift of zeitgebers and circadian period: shorter periods were associated with larger phase advance shifts and longer periods with larger phase delay shifts, a relationship not previously demonstrated in humans or within any other species. These findings have implications for understanding and treating the “disorders” produced when modern civilization forces an inappropriate timing of sleep, work and meals relative to the internal circadian rhythms, as occurs with shift work, jet travel and early school start times. These cases of circadian misalignment could be reduced or eliminated by appropriate circadian rhythm phase shifts.

Our species evolved in East Africa, around the equator, where the photoperiod (the duration of daylight) is relatively constant throughout the year. There are 12 hours of light and 12 hours of dark (LD 12:12) year round or LD13:11 when including civil twilight^26^. We speculate that the early humans who migrated north and eventually into Europe developed a longer circadian period because it was somehow adaptive for entrainment to the photoperiod that changed with seasons. Pittendrigh & Daan^27^ proposed that a period longer than 24 h, which is characteristic of diurnal animals, helps them keep an appropriate phase angle relative to dawn, whereas a period shorter than 24 h, characteristic of nocturnal animals, helps them track dusk. Latitudinal clines in circadian period have been found in insects and plants^26^,^28^. Presumably, there was no selection pressure to change circadian period for the humans who migrated into West Africa, because they were still living around the equator. Africans from western Africa around the equator were captured and sold into slavery to work on American southern plantations starting about 400 years ago^29^. Most African Americans are descendents of these people, and we speculate that they arrived in America with the original African circadian period. On the other hand, the immigrants from Europe had lived in Europe for thousands of years before coming to America. Since the recent arrival (in evolutionary terms) of Africans to America, there has been admixture with other ethnic groups in North America such as Europeans and Native Americans. The average European ancestry in our African-American subjects was 14%, whereas the Native American (Indigenous American) and East Asian ancestries were smaller, about 5%. Other studies, with similar methods for biogeographical ancestry estimates, have found similar proportions of European ancestry in African-Americans (13 to 20% in 6 northern cities and 12 to 23% in 3 southern cities^29^, 18% in Columbia, South Carolina^30^, 17% in Jackson, Mississippi^31^, 19% in Washington, DC^32^, 20% in the San Francisco bay area^33^, and 16% in Farmington, Connecticut^34^). The admixture in our sample of African-Americans could account for the overlap in circadian period length (Fig. 2).

Our finding of a shorter free-running circadian period in African-Americans compared to European-Americans confirmed our previous report^25^, but the difference was even larger. In the current study circadian period was 24.07 h for African-Americans and 24.36 h for European-Americans, a difference of .29 h, and in our previous study it was 24.18 h and 24.37 h, a difference of .19 h. This descriptively larger difference in circadian period could be because we asked our potential subjects about the races and ethnicities of their parents and grandparents and confirmed the assignment to groups with DNA samples. One participant completed the study, but when we received the results of genetic ancestry back we found that he was 49% European and 46% Sub-Saharan African, so we could not include him in either the European-American or African-American group. For more information about him see Supplementary Discussion 1.

There have been several studies of circadian period in humans (see Eastman et al.^25^, for a review) but we do not know of any that examined racial differences besides ours.

Duffy et al.^35^ found a sex difference in circadian period; it was 6 minutes shorter in women compared to men. We did not find a sex difference in circadian period, but our study suggests that sex differences depend on race. In European-Americans, a shorter circadian period was seen in women compared to men (9 minutes shorter), but in African-Americans a longer circadian period was seen in women compared to men (5 minutes longer). Our results for European-Americans and Duffy et al.'s agree in that women had a shorter circadian period. The 6-minute difference that they found was statistically significant, whereas the 9-minute difference we found was not (by a t-test). They had an extraordinary large sample size for this type of intensive human circadian rhythm laboratory research (105 men and 52 women), whereas our sample was much smaller (9 European-American men and 8 European-American women). Power analysis using our largest SD (.22 h) shows that we would need sample sizes of 34 per group for our 9 min difference to reach statistical significance. The difference in circadian period, with African-Americans having a shorter circadian period than European-Americans (17 min shorter), is much larger than the sex difference that Duffy et al.^35^ reported, with women having a shorter period than men (6 min shorter). If they had more African-American women than African-American men in their sample, then this could have helped to make the average period for women shorter compared to the men's. Future studies of the human circadian period should take race/ancestry into account.

It has long been known that there is a strong correlation between the phase angle of entrainment to the 24-h day and the free-running circadian period in lab animals^27^,^36^,^37^ and this has more recently been shown in humans^38^,^39^,^40^. We also found a significant correlation between circadian period and phase angle (Fig. 3) showing that individuals with shorter circadian periods had earlier circadian rhythms relative to dark, and those with longer circadian periods had later circadian rhythms relative to dark. Since the phase angle of entrainment depends on circadian period and circadian period is shorter in African-Americans compared to European-Americans, we would expect African-Americans to have earlier circadian rhythms relative to dark than European-Americans. We found the expected difference, with the rhythms of African-Americans being 0.5 h earlier (Table 2), but this difference was not statistically significant, likely because of the overlap in circadian periods between European and African-Americans.

People with early circadian rhythms relative to dark, or relative to local time, are called morning types or early birds, and people with late circadian rhythms are called evening types or night owls^38^,^41^,^42^,^43^,^44^. It has been proposed that the range of human phase angles of entrainment has increased to include more extremely long and more extremely short phase angles, because of the weaker zeitgebers of the modern world^44^. In our modern society, the extremely late chronotypes, the night owls, are usually sleep deprived on weekdays, because they have to wake up earlier than would be natural to go to work or school. It's clear from the problems of night owls and people with the delayed sleep phase disorder that it is not easy to change one's phase angle of entrainment (aside from living outdoors without electric lights^45^). In our modern world, it is better to have the short circadian period that we acquired during our evolution in Africa because it helps to produce the more socially acceptable phase angle of the early bird.

About half of the European-Americans, but very few African-Americans, delayed, shifted in the wrong direction after the 9-h advance of zeitgebers (antidromic re-entrainment) (Fig. 4). This makes sense because longer circadian periods favor delays (Fig. 5) and European-Americans had longer circadian periods. Data from actual eastward flights with good measures of circadian phase show that delays (shifting in the wrong direction) are more likely the more time zones are crossed^46^,^47^,^48^,^49^,^50^. Shifting in the wrong direction has also been seen in laboratory studies with large abrupt phase advance shifts of the zeitgebers^7^,^9^,^12^,^13^. None of these actual flights or lab studies compared people of different races or ancestries.

Re-entrainment of the circadian clock to a 9-h phase advance is possible via a 9-h phase advance or a 15-h phase delay. If advances and delays occur at the same rate (e.g., 1 h/day), then it is obvious that it will take much longer to re-entrain via delays (6 days longer). However, if phase shifts occured much faster via delays than advances, e.g. 1.6 h/day for delays and 1 h/day for advances, then the time for complete re-entrainment would be the same (9 days). Directional asymmetry for phase shifting (faster shifts in one direction) is well-known^20^. Our protocol had 3 advanced sleep/dark episodes, which was not enough for any subject to achieve complete re-entrainment (a phase advance shift of 9 h or a delay of 15 h), so we cannot know for sure if there would be an advantage to delaying or to advancing in terms of achieving complete re-entrainment. In any case, there could be differences in how people feel, sleep and perform during the days of advancing compared to the days of delaying irrespective of how long it takes for complete re-entrainment. This needs to be investigated.

Many subjects did not shift very far and many hardly shifted at all, and this was true for both European and African-Americans (see Fig. 4). The same variability in the magnitude of phase shifts, with some subjects shifting a lot and others hardly shifting at all, is seen in many laboratory studies of phase advances, e.g. Refs. 12, 13, 17, 51, and after real flights east, e.g. Refs. 46, 49. It is possible that we would have seen larger phase shifts if subjects were isolated from Chicago time when they were living on Kenya time during the phase advancing part of the protocol. With real jet travel people can have access to home time and communicate with family and friends at home, so our study mimicked real jet travel in that regard. It is possible, however, that the knowledge of clock time in Chicago was a conflicting stimulus to the circadian clock despite the LD cycle, which is considered to be the most important zeitgeber, being completely shifted. Subjects did not have access to windows and were confined to the lab. We might have seen larger phase shifts if subjects actually flew 9 time zones east to Kenya and were exposed to the natural LD cycle. However, such light exposure can be erratic and ill-timed, so that rhythms do not shift (see theoretical example, Fig 30-4 in Revell and Eastman^52^). We have shown that conflicting bright light can severely hinder the phase advance shift required after an abrupt phase advance shift of the sleep (dark) schedule^11^. Cases of travelers shifting very little, or not at all, after flying east and being in the new time zone for many days have also been documented^46^. Theoretically, controlling exposure to light and dark can hasten re-entrainment and reduce jet lag after flying across time zones^52^.

African-Americans had slightly smaller phase shifts in both directions (absolute phase shift in Table 2), than European-Americans. Appropriately timed bright light and melatonin can increase the magnitude of phase shifts^12^,^53^,^54^,^55^,^56^,^57^, so these treatments could be especially important for African-Americans. Studies are needed to determine if there are racial differences in how well these stimuli work to enhance circadian rhythm phase shifts.

In contrast to the well-established correlation between phase angle of entrainment and the free-running circadian period, we could not find any studies showing a correlation between the magnitude of phase shifts due to zeitgeber shifts and the circadian period within a species. Aschoff et al.^20^ deduced from oscillator theory that the rate of re-entrainment after phase shifts of the zeitgebers (and thus the magnitude of phase shifts) would be enhanced by a shorter period for advances and by a longer period for delays. This was supported by data from rats, who have a period >24 h, and re-entrained faster to a 6-h phase delay than a 6-h phase advance, and birds, who have a period <24 h, and re-entrained faster to a 6-h phase advance than a 6-h phase delay. Therefore, our finding (Fig. 5) that phase shift magnitude and direction was related to the underlying circadian period is novel, even though it is not unexpected.

## Methods

### Ethical Approval

The study was approved by the Rush University Medical Center Institutional Review Board and conformed to the standards set by the Declaration of Helsinki. Written informed consent was obtained from all subjects prior to their participation. Subjects were reimbursed for their participation.

### Subjects

Forty-two subjects were enrolled 3 to 4 days before the start of the 14-day laboratory study. Of these 42, 39 started the study, 38 completed the study and 36 had sufficient data to be included in the final analyses and are included in Table 1.

Subjects completed our Family/Ancestor Questionnaire and were asked to check all of the following categories that applied to them: White, Black or African-American, Asian, Hispanic or Latino, European, Middle Eastern, Far East Asian, Southeast Asian, Indian Subcontinent, North African, Afro-Caribbean, American Indian or Alaska native, Native Hawaiian or other Pacific Islander, Other, Don't Know. A short description was provided for each category. Subjects did the same for their biological mother, biological father, and their four grandparents. All the European-Americans in Table 1 endorsed White and/or European for all 6 of their relatives except for 2 subjects; one subject checked “Don't Know” for father and 3 grandparents and the other checked White and American Indian/Alaska Native for father and one grandparent. All of the African-Americans in Table 1 endorsed Black/African-American for all 6 of their relatives except for 5 subjects; 3 checked “Don't Know” for one grandparent, one checked both Black and White for one grandparent and one checked Black and American Indian/Alaska Native for self, mother and one grandparent. None of the subjects endorsed “Hispanic or Latino” for any of their 6 relatives.

Buccal (cheek) swabs were used to collect a DNA sample from each subject during the study, and were processed by Ancestry*by*DNA, DNA Diagnostics Center, Fairfield, OH. This company performed biogeographical ancestry estimates based on 176 ancestry informative markers, also known as population-specific alleles, which show large frequency differences between populations^58^,^59^. Results were returned several weeks later with percents for each subject in 4 categories (see Table 1).

Subjects were young, mostly in their 20 s, and healthy. They were not taking any prescription medications except for 4 women on oral contraceptives (2 African-Americans and 2 European-Americans). Due to the length of the study (14 days in the lab) most subjects were unemployed. Subjects were screened by telephone followed by an in-person interview and several questionnaires. Exclusion criteria included body mass index (BMI) > 35 kg/m^2^, night shift work in the preceding month, smoking and excessive alcohol or caffeine consumption. Subjects were given urine tests for common drugs of abuse and nicotine, and were breathalyzed 3 to 4 days before starting the study and on days 1 and 7 of the study. Subjects completed the Munich Chronotype Questionnaire (MCTQ)^44^ and the Owl-Lark (Morningness-Eveningness) Questionnaire (MEQ)^60^ during the study (Table 1).

### Protocol

This study took place in the Biological Rhythms Research Laboratory in Chicago from Jan 2013 to May 2014. Subjects were run in groups of three, and there was usually a mixture of African and European-Americans in each group. A group of three subjects was run in all months of the year, one group per month, except June, July and August because lab space was not available in the summer.

**Figure 1:**
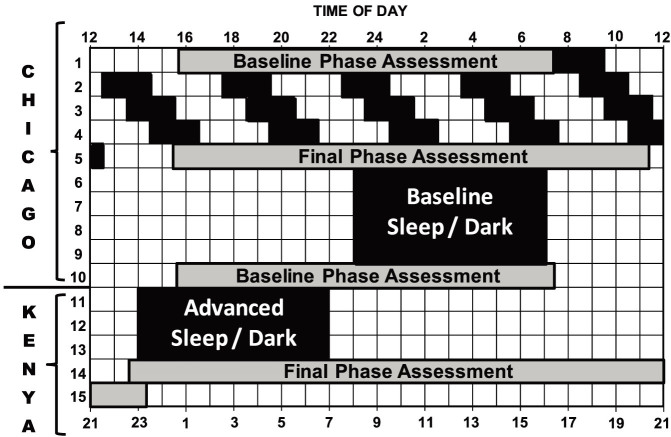
Protocol diagram. Subjects were in temporal isolation for the first 5 days (first 5 rows), and these days were used to calculate the endogenous free-running circadian period (τ). Subjects were put on an ultradian light/dark (LD) cycle for about 3 days (days 2–4). A 5-h LD cycle (LD 3:2) is shown. They were put to bed and permitted to sleep during the 2-h dark episodes and kept awake during the 3-h light episodes in relatively dim light (~10–30 lux). The ultradian LD cycle is a form of forced desynchrony during which the circadian clock free runs. The dim light melatonin onset (DLMO) was determined from 30 min saliva samples obtained during the circadian phase assessments (days 1, 5, 10 and 14), and was used as a marker for the phase of the master circadian clock. The phase shift of the DLMO from days 1 to 5 was used to calculate the circadian period. After the phase assessment on day 5, subjects were assigned an 8-h baseline sleep (dark) schedule similar to their sleep schedule at home before entering the lab. A 23:00 to 7:00 sleep schedule is shown (days 6–9). Subjects were given access to clocks, but we controlled their LD cycle (LD 16:8) and their time in bed, in the dark. After the baseline phase assessment on day 10, the sleep/wake schedule, LD cycle and meal schedule were advanced (made earlier) by 9 h for 3 days (days 11–13). The clocks in the subjects' bedrooms were also advanced 9 h, they were changed to Kenya time; Kenya is 9 time zones east of Chicago. The time line on the top shows Chicago time (noon to noon), and the line on the bottom shows the corresponding time in Kenya. The circadian phase shift of the DLMO from days 10 to 14 was used to determine the phase shift of the circadian clock due to the 9-h advance of zeitgebers (time cues).

**Figure 2:**
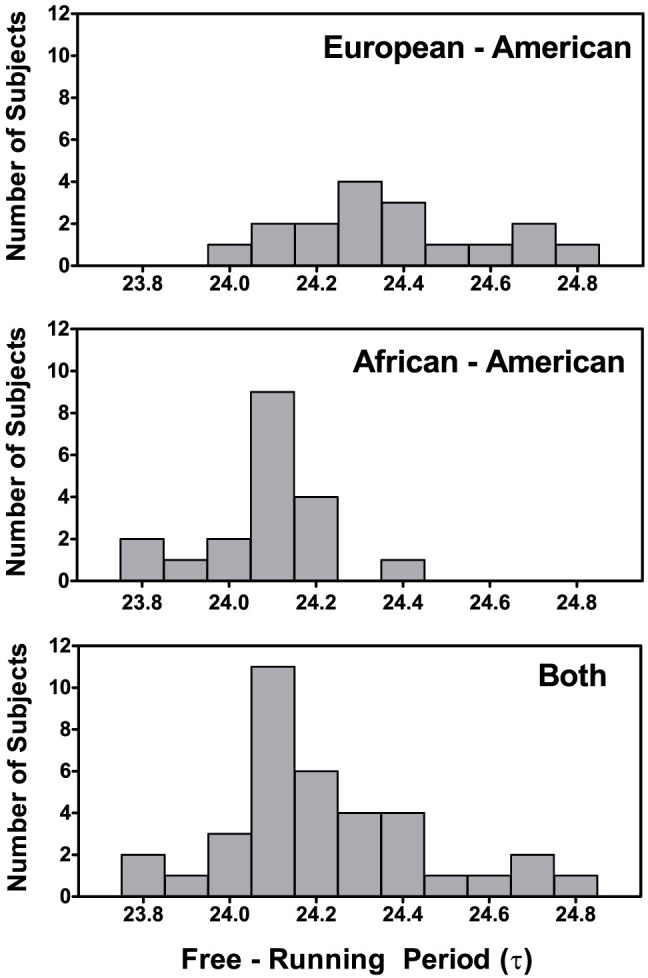
Frequency histograms of the endogenous free-running circadian periods (τ) for the European-Americans (N = 17), African-Americans (N = 19), and these subjects combined (N = 36). The free-running periods were calculated from the circadian phase assessments on days 1 and 5 (Fig. 1) which were before and after the ultradian light/dark cycles. The numbers on the x-axes show the midpoints of the bins, e.g., the 24.0 hour bin contains circadian periods from 23.95 to 24.04 hours.

**Figure 3:**
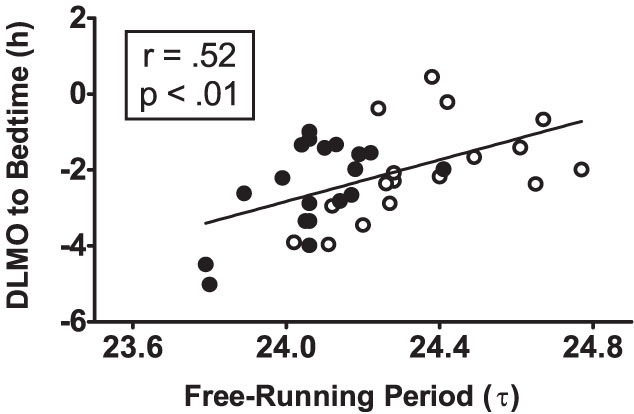
Scatter plot showing the relationship between the phase angle of entrainment of the circadian clock to the 24-h day during baseline and the free-running circadian period (τ). N = 36. Filled circles represent African-Americans (N = 19) and open circles represent European-Americans (N = 17). Phase angle of entrainment is the interval from the phase of the master circadian clock (assessed by the dim light melatonin onset (DLMO)) until dark onset (bedtime = lights out). The DLMO was determined from the phase assessment after the 4 baseline days. A negative DLMO to bedtime interval indicates that the DLMO occurred before bedtime, and a more negative number indicates a longer time from the DLMO until bedtime. Subjects with shorter free-running periods were entrained to the 24-hour day with earlier circadian rhythms (earlier DLMOs) relative to dark onset (relative to bedtime), and those with longer periods entrained with later circadian rhythms relative to dark. The diagonal line represents a linear fit of the data.

**Figure 4:**
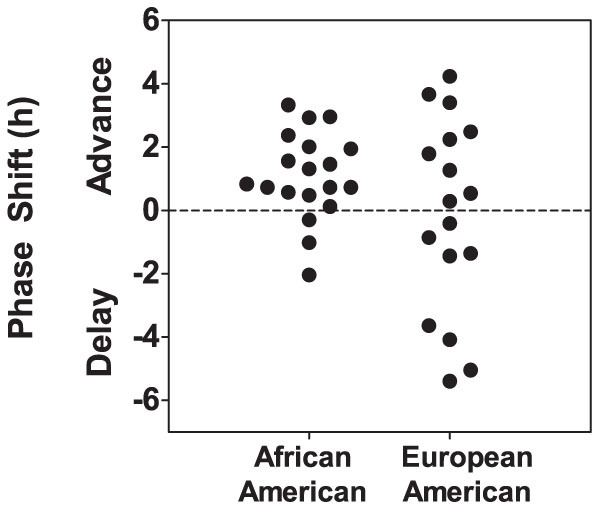
Phase shifts of the internal circadian clock due to the 9-hour advance of zeitgebers. Each dot represents the phase shift (from day 10 to 14 in Figure 1) of an individual subject. European-Americans (N = 17) were more likely to phase delay compared to African-Americans (N = 19); i.e. European-Americans were more likely to phase shift in the wrong direction.

**Figure 5:**
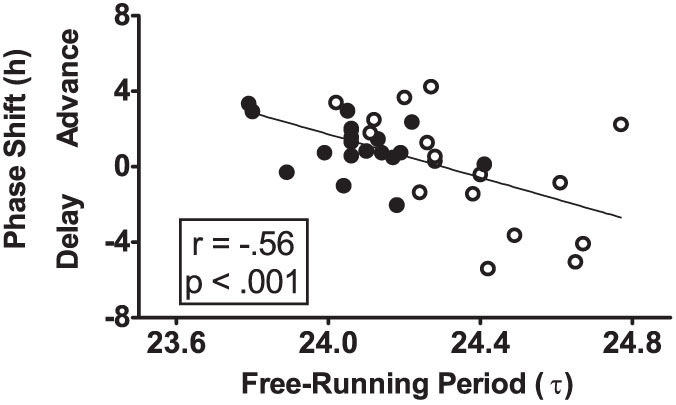
Scatter plot showing the relationship between the phase shift of the circadian clock due to the 9-hour advance of zeitgebers and the free-running circadian period (τ). N = 36. Filled circles represent African-Americans (N = 19) and open circles represent European-Americans (N = 17). Subjects with shorter circadian periods had larger phase advances, and those with longer periods had larger phase delays. The diagonal line represents a linear fit of the data.

During days 1 to 5 of the protocol (see Fig. 1), subjects were not given access to phones, lap tops, clocks, watches or any device that displays clock time. Their electronic items capable of time display were locked up from when they entered the lab on day 1 until day 6. The ultradian LD cycle was either LD 2.5:1.5 or LD 3:2 (Supplementary Methods, Ultradian LD Cycles). During the ultradian LD cycles subjects lived in a large, windowless, room that contained 3 large cots. The median light level was 19 lux (Supplementary Methods, Ambient Light Levels). Subjects ate and drank ad lib, but were not permitted caffeine or alcohol. Showers (1/day) were at random times. Subjects were required to remain in bed during the dark periods even if they could not sleep and were monitored by an infrared camera. While awake they sat around a large round table and ate, played games, read, watched pre-recorded movies and TV shows or engaged in other sedentary activities. After the phase assessment on day 5, subjects napped in the dark from noon to 4 pm.

Starting on day 6, subjects lived in the Bedroom Suite which has three bedrooms, a bathroom and a control room for research assistants. This was also a windowless environment and the bedroom and hallway lights were controlled by research assistants in the control room. Subjects had their own private bedrooms and were given their cell phones and any other electronics or watches they had brought with them (lap tops, tablets, etc). Baseline sleep schedules (with 8 h in bed, in the dark) were tailored to the individual using sleep diaries kept before entering the lab to best match the subject's natural sleep time. Each bedroom had one overhead fluorescent ceiling fixture on a dimmer switch. Each subject's bedroom fixture was set to its maximum for the first 10 h of the 16-h wake period, dimmed to the lowest level for the last 6 h, and turned off during the 8-h sleep episodes. The median light level was113 lux during the high intensity time, and it was 24 lux during the low intensity time (Supplementary Methods, Ambient Light Levels).

Meals were served at scheduled times starting when subjects were woken from the second baseline sleep episode. Breakfast was 1 h after waking, lunch was 5 h after breakfast and dinner was 6 h after lunch. In addition, subjects were allowed 2 small snacks per day of ≤160 calories. Caffeinated beverages and alcohol were not permitted. Each bedroom had a large wall clock set to Chicago time. When the LD cycle and the sleep schedule were advanced 9 h, the time of meals was also advanced 9 h to keep meals in the same phase relationship to the sleep schedule. The clock on the wall in each subject's room was changed to Kenya time, and a sign underneath the clock was changed from “Chicago” to “Kenya.” Each bedroom had a bulletin board where the subject's times for bedtime, wake time, and meals were posted. The times on these signs did not have to change when the LD cycle was advanced, because the new times were in Kenya time and matched the wall clock. As it was during baseline, the lights were set on high for the first 10 h of the wake period and on low for the last 6 h.

The dim light melatonin onset (DLMO), our measure of circadian phase, was assessed on days 1, 5, 10 and 14. Saliva samples were collected every 30 min in very dim light (<5 lux) using Salivettes (Sarstedt, Newton, NC, USA). Saliva samples were centrifuged, frozen, and later sent to SolidPhase, Inc. (Portland, Maine, USA) to be radioimmunoassayed (RIA) for melatonin. See Supplementary Methods, Circadian Phase Assessments.

Buccal (cheek) swabs for DNA were taken after subjects were awakened from the first baseline sleep episode, before they drank or ate anything. One hour after their wake up time they were allowed to leave the lab and go outside for 8 h. That was the only time during the entire 14-day study that they were not constantly supervised by research assistants 24 h a day. When they returned from this 8-h break they were given a urine drug screen to test for common drugs of abuse and nicotine, and were breathalyzed for alcohol. All subjects passed these screens.

### Data Analysis

Melatonin profiles were smoothed with a locally weighted least squares (LOWESS) curve set to medium, 10 points in the smoothing window (GraphPad Prism, GraphPad Software Inc., La Jolla, CA, USA). The threshold for determining the DLMO was 25% of the distance from the fitted minimum value to the fitted maximum value, i.e. minimum +25% (maximum - minimum). The threshold from the day 5 profile was used to determine the DLMOs on days 1 and 5, and the threshold from the day 14 profile was used to determine the DLMOs on days 10 and 14.

To calculate the free-running circadian period (τ) the difference between the DLMOs on days 1 and 5 was divided by 4 (because there were 4 days between these DLMOs) and then added it to 24 (when the DLMO delayed), or subtracted it from 24 (when the DLMO advanced). For example if the DLMO on day 1 was 21:00 and the DLMO on day 5 was 22:00, then the difference is 1.0 h. We divide this difference by 4 which equals 0.25 h. The circadian period is thus 24.25 h. If the DLMO on day 1 was 23:00 and the DLMO on day 5 was 22:00, then the circadian period would be 23.75 h.

To calculate the phase shift of the circadian clock due to the 9-h advance of zeitgebers the DLMO on day 14 (which was after the 3 advanced days) was subtracted from the DLMO on day 10 (which was after the 4 baseline days). Thus, if the DLMO on day 10 was 21:00 and the DLMO on day 14 was 19:00 then the phase shift would be +2.0 h. If the DLMO on day 10 was 21:00 and the DLMO on day 14 was 23:00 then the phase shift would be −2.0 h. By convention delays are indicated with a negative number.

We used t-tests to examine differences between African Americans and European Americans in the free-running period and phase shift. Given a previous report of a sex difference in circadian period^35^, a 2 (race)-by-2 (sex) analysis of variance was computed for circadian period. Pearson correlation coefficients were used to test for associations among the main outcomes measures. All reported tests are based on 2-tailed probabilities. Results are presented as means ± SD unless otherwise indicated. GraphPad Prism and SPSS (version 21) were used for data analysis.

## Author Contributions

C.I.E. conceived, designed and coordinated the study and wrote the paper. C.S. performed the majority of the data analysis and graphing of figures. V.A.T. performed data analysis and graphing of figures. S.J.C. performed data analysis and helped revise the paper.

## Supplementary Material

Supplementary Information Supplementary Information

## Figures and Tables

**Table 1:** Subject Demographics

	African American	European American
N	19	17
Sex	10 Men 9 Women	9 Men 8 Women
Age Range	21 to 43	22 to 40
Age (Mean ± SD)	33 ± 7.2	30 ± 5.7
MSF^a^ (Mean ± SD)	5.1 ± 1.7	5.2 ± 1.1
MEQ^b^		
Score (Mean ± SD)	55.0 ± 9.3	51.6 ± 5.2
# of M-Types	8	3
# of N-Types	10	14
# of E-Types	1	0
BMI (kg/m^2^) (Mean ± SD)	25.2 ± 4.6	24.6 ± 3.9
Genetic Ancestry^c^ (Mean ± SD)		
% European	14.4 ± 10.7	88.0 ± 9.1
% Sub-Saharan African	75.7 ± 11.2	3.1 ± 4.2
% East Asian	5.4 ± 8.0	2.7 ± 6.7
% Indigenous American	4.6 ± 6.5	6.1 ± 6.9

^a^Mid Sleep on Free Days from Munich Chronotype Questionnaire (MCTQ).

^b^Morningness-Eveningness Questionnaire (MEQ) score and number of Morning, Neither and Evening Types.

^c^Biogeographical ancestry estimates based on 176 ancestry informative markers.

There were no significant differences between the African-Americans and the European-Americans in age, MSF, MEQ or BMI.

**Table 2:** Circadian period (τ), baseline circadian phase, sleep schedule and phase angle of entrainment, and phase shifts to 9-h advance of zeitgebers. Mean ± SD unless otherwise indicated

	African American	European American
N	19	17
Free-Running Period (τ) (h)	24.07 ± .15	24.36 ± .22***
Baseline Circadian Phase^a^ (h:min)	21:38 ± 2:10	22:23 ± 1:19
Bedtime^b^ (h:min)	0:07 ± 1:40	0:25 ± 1:04
Waketime (h:min)	8:07	8:25
Phase Angle of Entrainment^c^ (h)	−2.5 ± 1.2	−2.0 ± 1.3
Number who Advanced^d^	14§	8§
Number who Delayed^d^	2§	7§
Absolute Phase Shift^e^ (h)	1.4 ± 1.0	2.5 ± 1.7*

*p < 0.05 by t-test.

***p < 0.0001 by t-test.

^§^p < 0.05, by Chi-Square test.

^a^The dim light melatonin onset (DLMO) from a phase assessment after the four 8-h baseline sleep episodes in the lab.

^b^Scheduled baseline bedtime (dark onset). Scheduled wake time (light onset) was always 8 h later.

^c^The phase angle of entrainment of the circadian clock to the 24-h zeitgebers during baseline, calculated as the interval from the baseline DLMO to baseline bedtime (dark onset). Negative numbers indicate that the DLMO occurred before bedtime.

^d^Number of subjects whose circadian phase (assessed by the DLMO) advanced or delayed by more than 0.5 h after the 9 h advance of zeitgebers.

^e^Phase shift of the circadian clock (assessed by the DLMO) after the 9 h advance of zeitgebers averaged without regard to sign (negatives entered as positives).
