# Supplementary material for: Circadian rhythm phase shifts and endogenous free-running circadian period differ between African-Americans and European-Americans
Source: Sci Rep. 2015 Feb 11;5:8381. doi: 10.1038/srep08381 (PMC4323653; doi:10.1038/srep08381)
Supplement: Supplementary Information [file srep08381-s1.pdf]

Supplementary Information

Circadian rhythm phase shifts and endogenous free-running circadian period differ between African-Americans and European-Americans

Charmane I. **Eastman**, Christina Suh, Victoria A. Tomaka, & Stephanie J. Crowley

Supplementary Table S1. Pearson correlation coefficient matrix for circadian variables (N=36).

	Baseline Bedtime	Baseline Phase	Phase Angle	Phase Shift
Baseline Phase[a]	+.76***			
Phase Angle[b]	+.01	+.65***		
Phase Shift[c]	+.03	-.35*	-.60***	
Free-Running Period (τ)	-.01	+.33*	+.52**	-.56***

* p < 0.05, ** p < 0.01, ***p <0 .001

[a]Baseline circadian phase assessed by the dim light melatonin onset (DLMO) from a phase assessment after the four baseline sleep episodes in the lab.

[b]Phase angle of entrainment during baseline; the interval from the baseline DLMO to baseline bedtime or dark onset. Wider phase angle (more negative number) indicates that the DLMO was earlier relative to bedtime, earlier relative to dark.

[c]Phase shift due to the 9-h phase advance of zeitgebers. Phase delays are negative numbers.

Supplementary Table S1 shows, as expected, a large positive correlation (r = +.76) between baseline bedtime (which ranged from 22:00 to 04:00) and baseline phase (DLMOs ranged from 19:20 to 02:40).

The significant correlation between baseline circadian phase and phase angle (r = +.65) shows that subjects with earlier circadian phases in Chicago clock time (earlier DLMOs) had earlier circadian phases (earlier DLMOs) relative to dark onset (bedtime), and those with later circadian phases in local clock time had later circadian phases relative to dark (bedtime). In other words, subjects who were morning-types relative to the artificial LD cycle of the lab were also morning types relative to the zeitgebers that exist outside the lab, and subjects who were evening types in the lab were evening types relative to outside world. This correlation held for both African-Americans (r = +.65, p<0.01) and European-Americans (r = +.66, p<0.01).

The correlation between phase angle of entrainment and free-running circadian period (r = +.52) is shown in Fig 3 and the correlation between phase shift and circadian period (r = -.56) is shown in Fig 5.

There were small but significant correlations between baseline phase and the free-running period (r=+.33, earlier DLMOs associated with shorter circadian periods) and between baseline phase and phase shift (r=-.35, earlier DLMOs associated with larger phase advances), but the correlations were larger when baseline phase angle was used instead of baseline phase (r=+.52 and r=-.60).

Supplementary Discussion 1

One participant completed the study, but when we received the results of genetic ancestry back we found that he was 49% European, 46% Sub-Saharan African, 5% East Asian and 0% Indigenous American, so we could not include him in either the European-American or African-American group. He endorsed Black for himself, his parents and all his grandparents on our Family/Ancestor Questionnaire. He had hazel eyes which we were not using as an exclusion criterion. Interestingly, dark skin and blue eyes are not uncommon in Cape Verde, West Africa.[1] This subject had a long circadian period, so if he was included in the African-American group it would have slightly decreased the difference in average circadian period between the European and African-Americans. On the other hand, he made the correlation between the proportion of European ancestry and circadian period in the African-Americans much larger (r = +.72, p<0.001 compared to r = +.49, p=0.037).

Supplementary Methods

Ultradian LD Cycles

A 4-h ultradian LD cycle (LD 2.5:1.5) was used to determine the circadian period for 10 subjects (6 African-Americans and 4 European-Americans) and a 5-h ultradian LD cycle

(LD 3:2) was used for the remaining subjects. Circadian period did not differ between the 4-h and 5-h ultradian LD cycles [$F(1,32)=1.29$, $p=0.27$] and a race-by-LD cycle interaction was non-significant [$F(1,32)=0.18$, $p=0.67$] indicating that the ultradian LD cycle length did not influence circadian period. The average circadian period from the 5-h LD cycle was 24.09 ± .15 h for the African-Americans and 24.39 ± .24 h for the European-Americans.

Ambient Light Levels

During the ultradian LD cycles overhead fluorescent fixtures on dimmers were locked to the lowest level. Light intensities were measured frequently, at each subject's eye, at the angle of gaze, with an Extech Model EA31 digital light meter. The research assistants took readings when subjects were in all the possible various positions in the room. Analysis of 1075 measurements showed that light levels were < 50 lux 98.4% of the time, and < 40 lux 93.1 % of the time. The median light level was 19 lux.

When subjects lived in the Bedroom Suite, the research assistants took readings when subjects were in all the possible various positions in the bedrooms and hallway. Analyses of 759 measurements showed that during the high intensity hours light levels were most often between about 50 and 400 lux, and during the low intensity hours they were between about 10 and 60 lux. The median level was113 lux during the high intensity time, and it was 24 lux during the low intensity time.

Circadian Phase Assessments

During the phase assessments subjects sat in La-Z-Boy recliners and usually watched pre-recorded movies and TV shows. Subjects were permitted to eat and drink ad lib, but no food or drink was permitted in the 10 min before each sample. Saliva samples were centrifuged, frozen, and later sent to SolidPhase, Inc. (Portland, Maine, USA) to be radioimmunoassayed (RIA) for melatonin. Each individual's samples were analyzed in the same batch. The sensitivity (limit of detection) of the assay was 0.9 pg/ml. Intra-assay coefficients of variation for low (daytime), medium (evening) and high (nighttime) levels were 20.1, 4.1 and 4.8% respectively. The inter-assay coefficients of variation for low, medium and high levels were 16.7, 6.6 and 8.4%, respectively.

References

1. Beleza, S. et al. Genetic architecture of skin and eye color in an African-European admixed population. *PLoS Genet* **9**, e1003372 (2013).
